# A Joining Procedure and Synchronization for TSCH-RPL Wireless Sensor Networks

**DOI:** 10.3390/s18103556

**Published:** 2018-10-20

**Authors:** Jose Vera-Pérez, David Todolí-Ferrandis, Salvador Santonja-Climent, Javier Silvestre-Blanes, Víctor Sempere-Payá

**Affiliations:** 1 Instituto Tecnológico de Informática (ITI), Valencia 46022, Spain; dtodoli@iti.es (D.T.-F.); ssantonja@iti.es (S.S.-C.); jsilves@disca.upv.es (J.S.-B.); vsempere@dcom.upv.es (V.S.-P.); 2 DISCA, Universitat Politècnica de València (UPV), Valencia 46022, Spain; 3 DCOM, Universitat Politècnica de València (UPV), Valencia 46022, Spain

**Keywords:** WSN, synchronization, IoT, IIoT, TSCH

## Abstract

Wireless Sensor Networks have become a key enabler for Industrial Internet of Things (IoT) applications; however, to adapt to the derived robust communication requirements, deterministic and scheduled medium access should be used, along with other features, such as channel hopping and frequency diversity. Implementing these mechanisms requires a correct synchronization of all devices in the network, a stage in deployment that can lead to non-operational networks. The present article presents an analysis of such situations and possible solutions, including the common current approaches and recommendations, and proposes a new beacon advertising method based on a specific Trickle Timer for the Medium Access Control (MAC) Time-Slotted Channel Hopping (TSCH) layer, decoupling from the timers in the network and routing layers. With this solution, improvements in connection success, time to join, and energy consumption can be obtained for the widely extended IEEE802.15.4e standard.

## 1. Introduction

Nowadays, the concept of the Internet of Things (IoT) is awakening great interest and achieving great popularity in the scope of emerging technologies. IoT is at the core of many innovative applications and services supporting digital transformation, and takes advantage of the ever-increasing number of connected devices. This set of technologies encompasses a multitude of scenarios and applications, such as Smart Cities, wearables, smart agriculture, health services, and vehicle-to-vehicle communication. It is also a key enabling technology for the so-called Industry 4.0 revolution (IoT for industry or IIoT) [1]. Inside the Internet of Things’ bag of assets, wireless sensor networks (WSN from now on) are fundamental for those digitalization services that require the agile deployment of sensing units, and network scalability and flexibility, in terms of reconfiguration and reorganization.

The context in which this study is based is the development of a “Deploy & Forget” tool to facilitate the tasks of installing and operating a WSN in industrial scenarios and address the need to streamline the initial stages of synchronization and connection to the network. The objective of this tool is that personnel without prior knowledge of communications are able to deploy a WSN without requiring external tools or qualified assistance, thus facilitating the adoption of wireless systems in industrial sectors.

Devices belonging to a WSN are characterized by constrained computing capabilities and power limitations, and are often battery-powered. Due to these characteristics, different protocols and standards have been proposed to implement low consumption communication systems. Among them, the IEEE 802.15.4 standard stands out, which serves as a base for other technologies that are commonly found in the industrial field, such as WirelessHart or ISA100.11a.

The IEEE802.15.4e amendment of this standard defines a medium access mechanism called Time-Slotted Channel Hopping (TSCH), which targets aggressive scenarios, such as industrial environments. TSCH allows us to perform frequency channel jumps between different transmissions to mitigate the heavy interference from this type of scenario. However, although the mechanism that is used by TSCH is very effective in steady-state operation situations, reducing losses due to interference considerably, it also hinders network-joining and first synchronization tasks. A node, a priori, does not know the time instant nor the frequency in which the synchronization beacons (carrying the information necessary to join the network) are being transmitted. For this reason, it is necessary to perform a scanning process, listening to different channels to detect and receive these synchronization messages.

In addition to the inconveniences that arise in the synchronization process using the TSCH mechanism, devices need to obtain information about the network topology once they have been synchronized. In order to choose the paths for routing the information to a WSN border router, this connection stage should be formed by a first phase of synchronization and a second phase of learning and connecting to the network topology. Therefore, a correct synchronization does not traduce directly or instantly in a node’s joining successfully to the network. For this second part, the IPv6 Routing Protocol for Low-Power and Lossy Networks (RPL from now on), as a de facto standard in terms of routing protocols for low consumption sensor networks, has been used. This combination of protocol (RPL) and standard (IEEE802.15.4e) has been widely extended in implementations of WSNs in industrial scenarios, although it still a subject of study as it can potentially be improved.

In order to overcome these drawbacks, a deep study has been carried out on the different parameters that intervene in the joining process and the topology definition in RPL. The results that were achieved, especially with the common configurations found in Request For Comments (RFCs) and WSN Operating Systems (OS) implementations, show room from improvement: sending more beacons means that the entire network synchronizes and connects much faster, but consequently has a much higher consumption, while reducing the number of beacons can render the network virtually useless. For this reason, a mechanism based on the trickle timer but independent of RPL has been defined. This allows for a reduction in the joining times in addition to maintaining a reduced duty cycle, which saves energy.

The complete protocol stack of the WSN has been designed as follows: the IEEE 802.15.4 standard as a physical level and MAC together with the TSCH medium access method, 6LowPAN as an intermediate layer to adapt the IPv6 frames so they can be transmitted over IEEE 802.1.5.4, and RPL as a dynamic routing protocol.

The rest of the article is structured as follows: in Section 2, a brief summary description is made about the IEEE 802.15.4 standard, the TSCH access method, and the RPL dynamic routing protocol. Section 3 shows related work and references addressing the problem of synchronization in WSN, the process of detection and joining the network, and processes that are related to the scanning phase of signalling beacons in the IEEE 802.15.4 TSCH standard. Section 4 details in depth the network synchronization and joining mechanisms that are tested and the modifications that are implied by its use with the RPL routing protocol. In Section 5, the testbed, experiments, and simulations are presented, while the results are analysed in Section 6. To close the article, Section 7 offers conclusions and introduces future work.

## 2. Overview on the IEEE 802.15.4 TSCH and RPL

Regarding WSN, there are different standards that allow for the construction of this type of ad-hoc mesh network. However, the IEEE 802.15.4 standard has managed to position itself as the de facto standard for this type of network [2], having provided inspiration for different industrial systems, such as WirelessHART [3], ISA100.11a [4], WISA [5], and ZigBee [6]. In the same way, the RPL protocol has been proposed as a standard solution for the routing layer to build paths towards a root node (convergecast traffic). There is a great variety of publications that are related to this routing protocol, and provide different improvements and modifications. These solutions are mainly based on obtaining different routes according to the criteria that are defined in the objective functions (OF) used by this protocol. The main aspects of the aforementioned protocols are summarized below.

### 2.1. Time-Slotted Channel Hopping

Initially, the medium access method of the IEEE 802.15.4 standard [7] allowed for establishing communications through guaranteed slots or contention-based periods using a Superframe structure that was delimited by beacons. Following the IEEE 802.15.4e amendment of 2012, some improvements that were proposed by a proprietary mechanism were added [8], and other channel access methods were defined [9], among which the Time-Slotted Channel Hopping (TSCH) method, which is focused mainly on industrial environments, stands out.

The TSCH access method is part of the MAC mechanism set of the IEEE 802.15.4e amendment of the standard. Among all of the methods that were included, TSCH is specifically designed to work in aggressive environments where interference and fading hinder communications in the sensor network. This mechanism replaces the concept of Superframe with the Slotframe, which is nothing more than a succession of time slots that are repeated at certain intervals. An in-depth description about the TSCH method’s timeslot timing template can be found in the standard [7]. In addition, it adds frequency diversity thanks to its channel-hopping mechanism, which allows for mitigation of the effects due to interferences. These characteristics provide greater robustness, as well as work cycle optimization, thanks to the use of optimal radio resources planning both in the time domain and the frequency domain.

**Figure 1:**
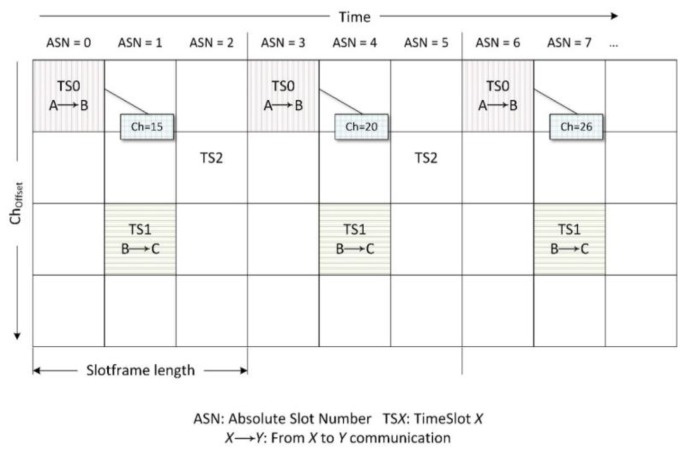
The timeslot and channel offset matrix used in the Time-Slotted Channel Hopping (TSCH) method.

Figure 1 illustrates an example of a timeslot and channel offset schedule. Communication between a pair of devices is scheduled during the same timeslot and channel offset, and repeated every slotframe. Channel offset provides an opportunity to use the same slot in the schedule with a different frequency at each iteration of the slotframe. The channel used to establish a connection in each of these slots changes according to Equation (1):

(1) $$\;f=F\left\{\left(ASN+ch_{offset}\right)mod\;C\right\}\;$$

where f is the physical transmission channel; ASN is the absolute slot number, which corresponds to the number of slots that have passed since the network was deployed; the $ch_{offset}$ parameter is a parameter that allows for different channels to be used in the same time slot; and C corresponds to the total number of channels that are used. Therefore, the function F{−} consists in selecting a channel of a predefined sequence according to the index that is obtained after calculating the module.

The configuration and the steps that are required to obtain an efficient schedule are outside the scope of the IEEE 802.15.4e standard. The chosen implementation during the experiments is the autonomous scheduler Orchestra [10]. Orchestra provides the opportunity to maintain multiple schedules, each allocated to a particular traffic type, with different sizes and timeslots assigned to them. Orchestra allows the nodes to autonomously obtain a simple schedule and update it automatically as the topology evolves and is fed with network information by the RPL layer.

#### TSCH Scanning and Synchronization Procedures

A synchronization phase is first required due to the mechanism that underlies the operation of TSCH. This mechanism is based on the division of time into slots of sufficient duration so that two nodes can communicate with each other. By using such a short time slot to communicate, devices must maintain synchronization between themselves. For this, all devices that are part of the WSN must follow the same absolute time reference, which in this case is represented by the absolute slot number (ASN). This parameter represents the number of slots that have passed since the network was started. In addition, the nodes need to control the clock drift that can occur to keep this synchronization and the slots of several devices aligned in order to maintain the strict time limits that communication requires.

Nodes that wish to join the network must request the information from the nodes that are already part of the network. For this reason, Enhanced Beacons (EB) are used to broadcast information on synchronization, sending broadcast messages that contain, as their main form of information, the ASN value of the device itself to serve as a time reference to new devices.

Thus, the synchronized nodes will be broadcasting EBs periodically, while the nodes that wish to join will be listening for a considerably longer time, in comparison to the slot length, in different channels until they get a signalling message to correctly adjust their time origin.

### 2.2. RPL: The IPv6 Routing Protocol for Low-Power and Lossy Networks

**Figure 2:**
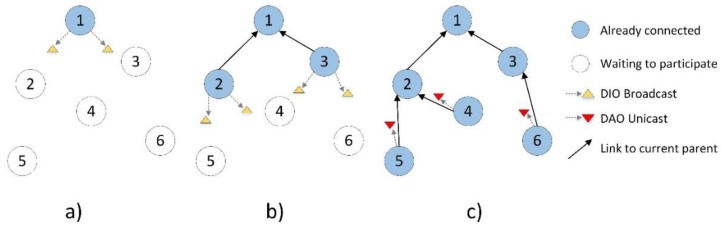
The exchange of Routing Protocol for Low-Power and Lossy Networks (RPL) control messages to build the topology. DIO, Destination-Oriented Directed Acyclic Graph (DODAG) Information Object; DAO, Destination Advertisement Object. (**a**) Sink node starts the dissemination of the RPL information with DIO messages; (**b**) Connected nodes forward the information downwards; (**c**) Connected nodes send upwards a DAO message.

The RPL protocol is specified in RFC 6550 [11], and is responsible for providing routing (including a route selection mechanism) from multi-point sources to a central node in addition to supporting point-to-point traffic. The protocol is designed for discovering and maintaining routes in devices with limited processing, memory, and energy capacities, which is why RPL has become widely used in WSNs. The topologies in RPL are represented by a Directed Acyclic Graph (DAG), a graph that organizes links to avoid loops. RPL provides the ascending routes of each node of the network to the root, forming a Destination-Oriented DAG (DODAG) that is optimized according to an Objective Function (OF). A DODAG’s formation and maintenance is done through different control messages: DODAG Information Solicitation (DIS) messages are used to request information about the RPL’s topology; DODAG Information Object (DIO) messages are sent periodically or in response to a DIS, using the Trickle Timer method, to disseminate information about the RPL’s topology; and Destination Advertisement Object (DAO) messages are propagated upwards to build downwards routes. The objective functions define how the RPL nodes must select and optimize the routes within the same RPL topology. In these definitions, you can find how the nodes transform one or more metrics into a certain rank, which symbolizes the approximate distance to the DODAG root. The construction of the DODAG is performed according to the procedure that is illustrated in Figure 2.

#### RPL DIO Transmission Using a Trickle Timer

For the transmission of DIO messages that are used in RPL for the dissemination of the information necessary to join the topology, a mechanism that is based on Trickle Timer, which is specified in RFC6206 [12], is used. This mechanism allows for the set of nodes that use a shared lossy medium to exchange information in a more energy-efficient and scalable way. The algorithm is responsible for modifying the transmission window of the DIO messages, reducing the quantity of control messages, as long as the information corresponding to the topology remains stable. In this way, a node transmits DIO messages unless it receives messages from other neighbours, whose content suggests that the information to be transmitted is redundant.

1. The configuration of the Trickle Timer mechanism is based on three basic parameters: IntervalMin ($I_{min}$), Interval Doublings (ID), and Redundancy Constant (RC). Taking into account these parameters, the Trickle Timer mechanism works in the following way:
2. When the algorithm is started, the current value of the timer is set to its minimum value, which is defined by the $I_{min}$ parameter, after which the first interval of this duration begins.
3. Whenever the node listens to a transmission that it considers “consistent” with the current topology, it increments a counter called  c.
4. When the timer expires, the node transmits if and only if the counter is less than the redundancy constant, which is defined as  RC.
5. When the timer expires, the algorithm doubles the length of the interval until it reaches the maximum value given by $\;I_{min}\cdot ID$, and remains in this setup until an event resets the timer.
6. If a node receives a transmission that it considers “inconsistent”, the node resets the Trickle Timer. To restart it, the node sets the timer back to $\;I_{min}$.

The description of this mechanism is important, since some implementations, such as the one performed by the Contiki OS [13], use the Trickle Timer mechanism to modify the Enhanced Beacon transmission period in addition to the transmission of DIO messages.

## 3. Related Work

Ever since the IEEE Computer Society released the IEEE 802.15.4e amendment in 2012, which included the TSCH mechanism, network synchronization has been a popular topic in Wireless Sensor Networks research. The following works [14,15,16,17] consider problems in synchronization due to inaccurate estimates of the deviation between two slots. To improve the synchronization process, adaptive synchronization mechanisms have been proposed, obtaining very accurate synchronizations, reducing traffic, and saving energy. Their solutions allow us to improve the drift clock estimations; however, no mechanism for advertising beacons that minimizes the network joining time has been developed. Duquennoy et al. [18] summarize the main challenges and difficulties that arise when using TSCH in Contiki, highlighting that very precise synchronization can be achieved with the current implementation.

In 2015, the Internet Engineering Task Force (IETF) presented an informative document [19] detailing some of the issues with, and objectives for, the TSCH mechanism, among which is the formation and maintenance of the network. The 6TiSCH working group has also been putting its best efforts into improving the mechanism to make it possible to use IPv6 in TSCH networks. A minimal mode of operation has been defined in [20], which specifies the recommended configuration of some protocols that are related to TSCH; for instance, the routing protocol RPL. However, a mechanism that improves the network joining process or the relationship between RPL and TSCH has yet to be proposed.

Vogli et al. propose and improve in [21,22] a series of mechanisms that improve the speed with which the nodes join the network, studying each of them using an analytical model. Duy et al. [23,24] carry out another analysis on the problems of long delays in connecting to the network and propose a new slotframe-planning scheme to improve the efficiency of the TSCH union mechanism. This solution allows a device to dynamically determine the number of synchronization messages that is needed.

Khoufi et al. [25,26] evaluate the time needed to form a TSCH-based network using the NS3 simulator, proposing a new algorithm for sending Enhanced Beacons. Kim et al. [27] propose a new scheme to join the network, based on channel quality estimates, that is more efficient in environments where interference is more severe.

The works described in [21,22,23,24,25,26,27] propose different solutions based on EB transmission schemes that improve synchronization times and enable advertising-based slots in the TSCH schedule. These solutions are based on statically improving the planning, and assume that with a higher number of synchronized nodes the synchronization is faster because there are more messages with information on synchronization. Although this is true, networks do not always have enough nodes to improve synchronization in this way.

De Guglielmo et al. [28] evaluate a TSCH announcement algorithm that is based on the random transmission of synchronization messages. Their results show that the joining time of a node is mainly influenced by the number of channels that is used for the transmission of beacons on the nodes that are already part of the network.

Wang et al. [29] evaluate the impact of different parameters during the joining phase, demonstrating the impact of EB transmission and their possible collision in large networks. The different simulations in this work consist of varying the density of nodes as well as the transmission period of the EBs, and the authors arrive at the conclusion that a random period manages to improve the connection times in denser networks.

Vallati et al. [30] is one of the few works that analyses problems with the joining time in TSCH networks while considering the entire protocol stack, including the routing protocol RPL. A comparison between a minimal configuration defined by 6TiSCH and a dynamic resource management algorithm has been proposed, showing the improvements that can be made to the transmission of EBs and RPL control messages during network formation.

In this work, a study is carried out to improve the synchronization process and the initial connection in a WSN, analysing the basic parameters that directly intervene in the synchronization phase, regardless of the number of nearby devices of the waiting/joining node. In addition, unlike the abovementioned studies, the nodes’ behaviour is analysed to obtain a fully deployed network, using TSCH to synchronize the devices and the RPL protocol for the construction of the topology and the creation and maintenance of routes. This is relevant because all of the traffic that circulates through the network during both the synchronization phase and the connection phase is taken into account, and allows us analyse the impact of the RPL protocol when used in combination with a time-synchronized and frequency-hopped access mechanism.

## 4. Joining Procedure and Time Synchronization Problems

As already mentioned, the TSCH medium access mechanism improves the reliability of communications considerably, since it is specifically designed to work in industrial environments where this type of interference hinders access to the radio environment. However, this is only valid for steady-state operation, since its behaviour during the initial moments of the network is more complicated when compared with other contention-based asynchronous mechanisms that use only one transmission channel.

Moreover, the RPL protocol has proven to be a fundamental tool for establishing communications and links in mesh networks with low capacities. However, if we analyse the behaviour of these two protocols (IEEE802.15.4e-TSCH and RPL) in early phases where synchronization has not yet been performed, it is difficult to form the network rapidly [14,16,17,24,28]. The missing factor in the operation of RPL and TSCH is that the beacon transmission period, which is necessary to carry out the synchronization, has been modified with the RPL Trickle Timer, which does not take into account the needs of the synchronization layer. For a node to be able to restart the transmission period of beacons and DIO messages, it is necessary for the node to receive a DIS message. However, DIS messages cannot be sent by those nodes that are not yet synchronized. For instance, in situations where the network is already stable, the new nodes that wish to join the network request information through DIS (DODAG Information Solicitation) messages, but when using TSCH, the new nodes are not yet synchronized and the transmission of DIS messages will not be possible since these messages are discarded. This is a key concept, as synchronizing successfully does not mean that the node is able to transmit data, as it does not know any routes.

Therefore, this initial deployment phase can be subdivided into two stages. In the first one, the nodes that wish to join the TSCH network begin to scan in all of the channels in search of the synchronization EBs that already-connected nodes are transmitting. Then, in the second stage, nodes have already synchronized (they know the ASN value of their temporal reference and the channels they must use in each slot), but still need to join the RPL network. These nodes start sending DIS messages to request routing information, while already-connected nodes reply or send periodic DIO messages with the required information. The following content of this section explains in detail these two processes or stages.

### 4.1. TSCH Network: Scan Phase and Enhanced Beacon Broadcasting

In the first stage, nodes that are already connected to the network send EBs periodically according to the EB Transmission Time (EBTT), while nodes that are waiting to synchronize are listening, sweeping the defined channels, during a scan time called TSCAN. The total number of channels is defined by the Number of Channels (NC); however, it is possible to have a total number of different channels for the scanning phase, which is determined by Number of Scan Channels (NSC).

**Figure 3:**
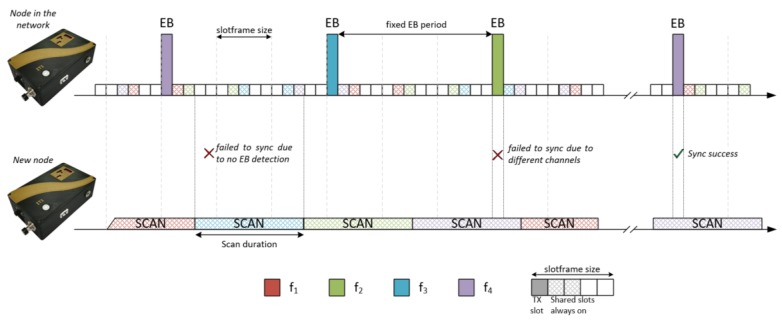
The TSCH method’s scan and synchronization phase. EB, enhanced beacon.

Figure 3 represents the two-node slotframes in this TSCH synchronization phase using four frequencies (f1 to f4). There are circumstances in which a node that is scanning does not locate any signalling message. Depending on the configuration of the parameters EBTT, TSCAN, NC, and NSC, it can be observed that the synchronization process can be easier or harder, since it is influenced by the amount of synchronization messages that are transmitted and by the listening time in each channel.

### 4.2. Exchange of RPL Control Messages

Once the incoming device is synchronized by TSCH, it can follow the same pattern of active and inactive slots within the slotframe. However, the node does not yet know about the topology of the network, as this requires it to receive an RPL DIO message. The node that is trying to join the topology will begin a process of sending DIS messages requesting the information necessary to join the topology. These messages will be transmitted with a fixed period that is determined by the DIS Transmission Time (DISTT). In contrast, the nodes that are already connected to the network are sending DIO messages using the Trickle Timer mechanism that was described in the previous sections.

**Figure 4:**
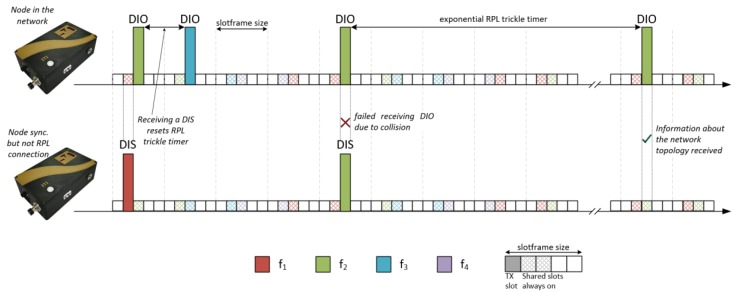
Exchange of RPL control messages. DIS, DODAG Information Solicitation.

Figure 4 represents the RPL message exchange mechanism after synchronization. It can be seen how the DIS messages that are received by nodes that are already part of the network restart the Trickle Timer, forcing DIO messages to be sent more frequently. The connection to the RPL DODAG only occurs when the new nodes receive the DIO messages with the topological information.

### 4.3. Enhanced Beacon Period Using the RPL Trickle Timer

This last configuration is featured in the Contiki OS when configuring a network that uses the routing protocol RPL along with the TSCH access mechanism. In this configuration, the TSCH mechanism relies on the RPL Trickle Timer to modify the transmission period of EBTT beacons. This modification avoids transmitting EBs with a fixed period, aiming to send less EBs when the network is more stable and a long period has passed since synchronization started. In this way, network congestion is considerably reduced, since when the network remains stable the number of control messages that is sent is significantly lower. However, the TSCH parameters are being modified based on the needs of RPL, which often results in much longer network connection times. It is necessary for the synchronized nodes to receive a DIS message in order to restart their Trickle Timer and send EBs more frequently. Nodes that are not yet synchronized begin the scanning process to receive beacons; however, these nodes are not able to send DIS messages since the messages are discarded at the MAC level as long as they are not synchronized. As introduced in the description of the Trickle Timer mechanism, the nodes do not restart this timer if they see that the network is circulating redundant information in the RPL control messages. This means that if a node does not receive any DIS messages, restarting the Trickle Timer on its own may take a long time, and no EBs will be sent in the interim, which greatly reduces the number of opportunities that new nodes will have to listen to synchronization messages.

**Figure 5:**
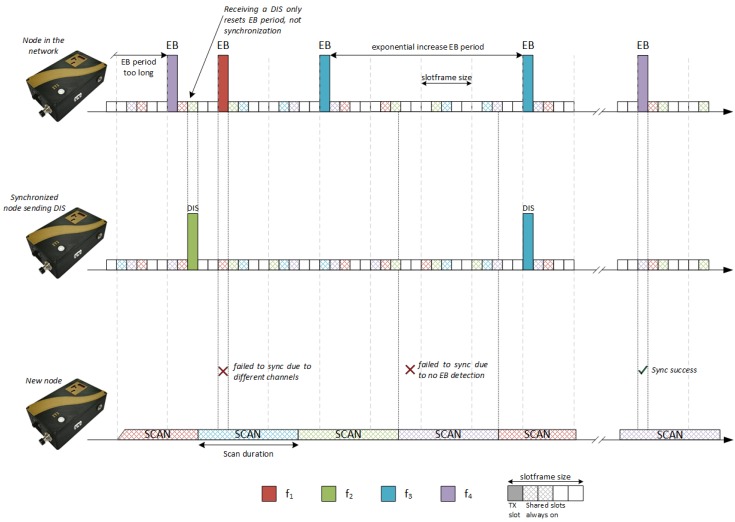
A combination of TSCH and RPL.

In this configuration, the Trickle Timer is used to modify the EBTT parameter; so, upon receiving a DIS message, nodes would be forced to be transmit more EB messages in addition to the DIOs. However, if the incoming node is not synchronized, DIS messages that can restart the Trickle Timer will not be sent. Only nodes that have synchronized but that have not obtained the RPL topology will be sending DIS messages. Figure 5 represents this configuration, in which the DIS messages are interspersed between the scan periods of the beacons.

### 4.4. Common Configurations, the Proposal, and Involved Parameters

The analysis of the network joining mechanisms offers a list of involved parameters that can affect, either positively or negatively, the process. Tuning a parameter in order to favour a desired trait—for instance, joining speed—can result in an impracticable amount of energy consumption.

Identified variables that can influence the results, and serve as input for different experiments and simulations, are:

- the number of channels used during transmission;
- the number of channels used for scanning the medium before synchronization;
- the EB (beacon) period, be it fixed or variable; and
- the RPL control message (DIS, DIO) period.

The control message traffic rate policies, which are represented by the EB and RPL periods, are commonly found under two configurations: the RPL trickle timer, as seen in Section 4.3, and the fixed EB period, as seen in Section 4.1. These are the options that are recommended by the Contiki OS and the IEEE802.15.4e standard with the minimal 6TiSCH RFC, respectively.

A proposal for a new configuration, based on the best traits of these two configurations and called “Custom Trickle Timer”, allows nodes to increase or decrease the EB period at a certain moment. A detailed description is introduced in the results section. Since many of the proposed configuration’s parameters are derived from the common configurations’ results, it is better to explain it after characterizing the performance of the “Fixed EB” and the “RPL trickle timer”.

## 5. Simulations and Testbeds

The different mechanisms that are frequently used for synchronization and routing are prone to show very different behaviours, which impact on the network’s formation time. Therefore, it is interesting to make a comparison of the different results in terms of the probability of synchronization/connection success, the amount of messages that are generated, energy consumption estimates of different devices, and above all the network’s synchronization and connection speed. 

**Figure 6:**
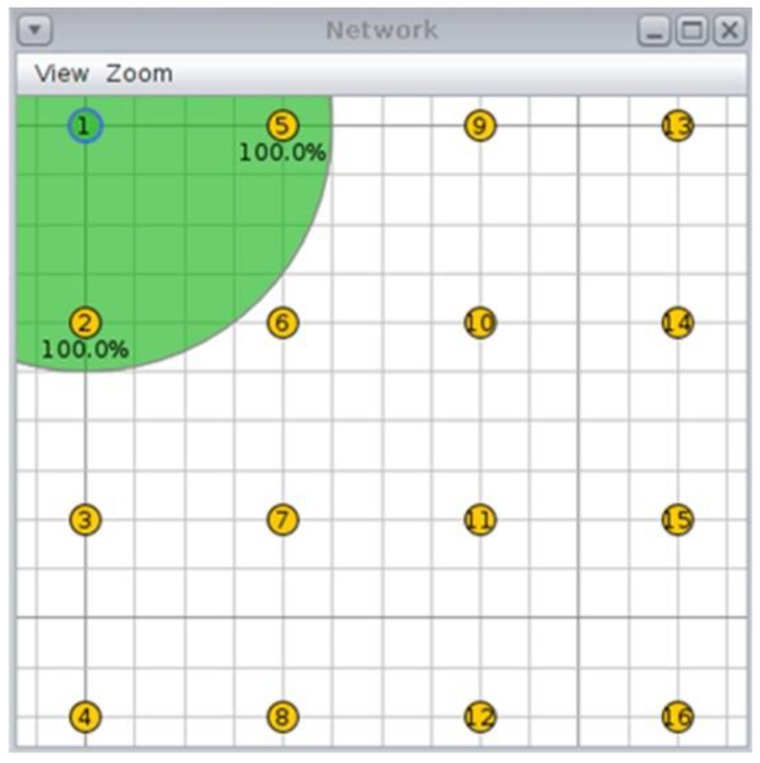
The physical topology.

Different simulations have been carried out for this purpose using the Cooja simulator that is included in the Contiki OS, which allows us to compare the operation of different parameter configurations. A network that is formed by a grid of 16 nodes has been defined, in which only one of them takes on the role of coordinator (node 1 in Figure 6). This node, which is located in the upper left corner, is responsible for initiating the synchronization process by disseminating the EBs and RPL control messages.

The configuration parameters for the simulation are shown in Table 1. In this topology, nodes are separated by a fixed distance of 40 m, forcing the creation of a multi-hop topology. This allows us to observe the impact of synchronization on the furthest nodes from the coordinator. The duration of the simulations is limited to 15 min, considering that the objective is to test the agility of the process; so, this amount of time is enough and longer times are not acceptable for deployment personnel. This limit matches the assumption that nodes need no more than 2 min for the next level to connect, thus needing 12 min for all six levels to connect correctly. For different topologies and scalability issues, longer simulation times may be required.

In order to have a point of reference for comparison, the behaviour of a configuration that uses TSCH as it is defined in the standard, with a fixed beacon transmission period (that is, the method described in Section 4.1 and Section 4.2), is simulated. The configuration that combines TSCH with RPL is also used to modify the beacon transmission period according to the result of the RPL Trickle Timer (that is, the method described in Section 4.3).

All simulations use Orchestra-based TSCH resource planning [10] in order to control the resources that are given to RPL individually. From these base configurations, different combinations of parameters that influence the synchronization and connection process are simulated.

The aim is to reach an optimal configuration that improves the synchronization and connection process and the time it takes to complete this process. Table 2 shows the parameters that have been modified to perform the following simulations.

For the energy consumption analysis, the parameters in Table 3 have been taken into account, corresponding to the consumption of the radio transceiver CC2420 datasheet, which is the hardware that is used by the Zolertia Z1 node model that was chosen to perform the different simulations.

## 6. Results

The following figures explain the results that were obtained by the previously described testbed and experiments. The different graphs show the probability of synchronization and connection success for each node in the network. In this way, it is possible to verify which configuration and which mechanism improve the process for joining the network.

In addition, the number of messages that are transmitted, which is directly associated with the mechanism of connection to the network, such as EBs or the control messages of RPL, has been also analysed. This study allows us to obtain an estimate of the energy consumption for each node. Other interesting results include the maximum time to connect all of the nodes in the topology, synchronized and connected by RPL.

**Figure 7:**
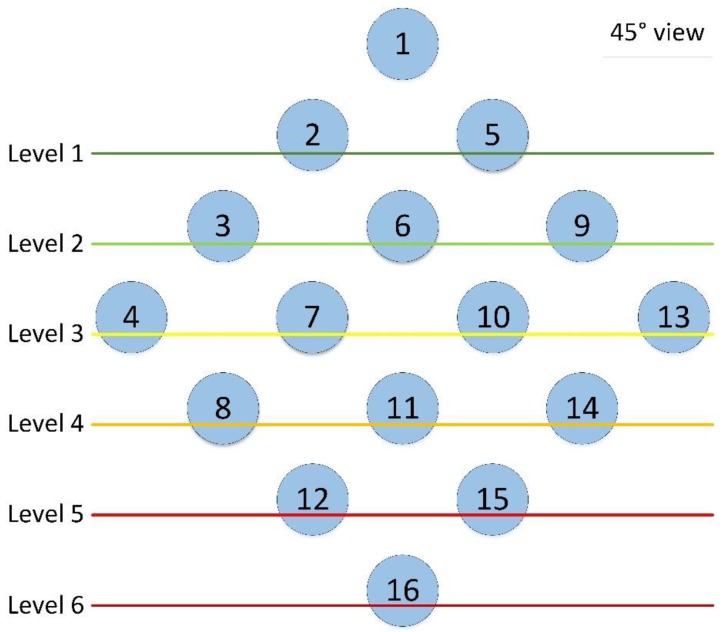
The propagation direction for each level.

Figure 7 illustrates the direction in which the synchronization should be propagated. For instance, until nodes 2 and 5 are connected, the synchronization cannot be propagated to the rest of the network.

### 6.1. Probability of Connection Success

**Figure 8:**
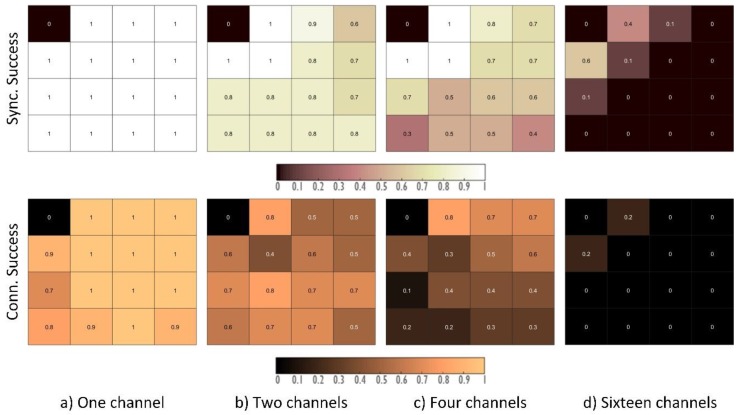
The probability of synchronization and connection success for different numbers of channels.

The first batch of results aim to represent the probability of a successful connection to the network. Figure 8 shows the comparison of four different configurations when changing the number of channels that are used to establish communications (Test 1 shown in Table 2). These graphs show a representation of the physical topology of the nodes and their synchronization/connection success probability in the 15 min that were taken as a reference during the experiments. Specifically, these four configurations use the EB transmission mechanism based on the RPL Trickle Timer that is described in Figure 5 and provided in previous releases of the Contiki OS (i.e., the method described in Section 4.1 and Section 4.3). Clearly, increasing the number of communication channels reduces the probability that a node is transmitting an EB in the same channel that another node is scanning. This behaviour can be observed, for instance, when 16 channels are used: more than 60% of the nodes have not connected after the 15-minute simulation.

A key observation is the impact of the RPL protocol in TSCH synchronization: when a node does not manage to establish a connection with its neighbours to become part of the topology, it does not start transmitting its own EBs, thus interrupting the propagation of synchronization to further nodes. This can be clearly seen in the configuration that uses a single channel. In this configuration, all the nodes managed to synchronize one of their neighbours; however, some nodes have not managed to join the RPL topology, thus damaging the nodes below.

On the other hand, in the configuration that uses 16 channels, it is possible to see the effect of synchronization propagation: as the connection probability decreases at each level (see Figure 7), the nodes in the following levels are unable to synchronize because no EBs are transmitted downwards.

**Figure 9:**
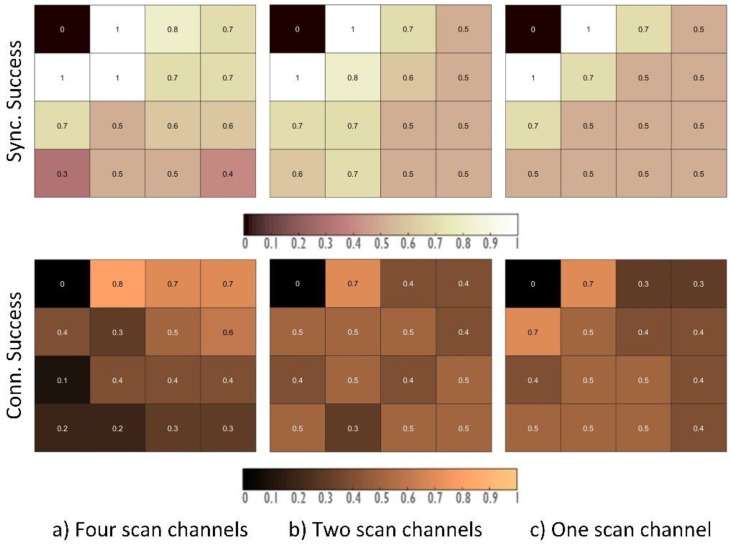
The probability of synchronization and connection success for different numbers of scan channels.

In spite of these results, a logical next step in the configuration is to use fewer channels only during the scanning phase (Test 2 shown in Table 2). Therefore, nodes that are already connected transmit signalling messages, among other data, using four channels, and the nodes wanting to join would only scan in a smaller number of these channels. Figure 9 shows the results for a configuration in which four channels are assigned for the stable operation of the network, but that uses a different number of channels for the scanning phase.

These three configurations use the Trickle Timer to modify the beacon transmission period. As can be seen, the connection probability slightly improves when using less channels when scanning; however, the improvement is not significant enough to consider it an optimal configuration.

Finally, to complete the configuration of the possible parameters that can lead to the optimization of the network joining procedure, the impact of the beacon transmission period has been analysed (Test 3 in Table 2), achieving interesting and practical results. Initially, the Trickle Timer was used to modify the beacon transmission period, as recommended in the Contiki implementation. However, this means that, once the network has started and some time has passed, not enough beacons will be sent since the Trickle Timer algorithm does not take into account the TSCH method’s requirements and is only reset by the RPL protocol. This leads to the inclusion of other configurations, such as leaving a fixed beacon transmission period (for instance, the period initially set by the standard recommendation, which can be found in the OpenWSN OS), or the addition of a beacon transmission mechanism that varies with time and is independent of RPL.

The proposed configuration, “Custom Trickle Timer”, is designed to address the issues that were detected in the previous tests, and consists in transmitting beacons every 4 s during the first 2 min. This time is chosen after verifying that it is the maximum needed to connect all of the neighbours within the coverage area of a node sending EBs. The probability of a node connecting after 2 min is negligible. Once these two minutes have passed, the beacon transmission period is changed to 16 s.

This stimulates the synchronization process over a short period, and then relaxes because the nodes in the next level, which are recently connected, will start sending beacons on their own with the 4-s interval. Hence, it takes advantage of the propagation behaviour of the synchronization, speeding up but keeping the energy and congestion sustainable.

In addition, this assumption results in 12 min for all six levels in the selected topology (Figure 7) to connect correctly, which is then successfully covered by the 15 min simulation time that was chosen.

As can be seen, increasing the number of beacons considerably improves the synchronization and connection mechanism’s success, achieving configurations that connect successfully virtually 100% of the time.

**Figure 10:**
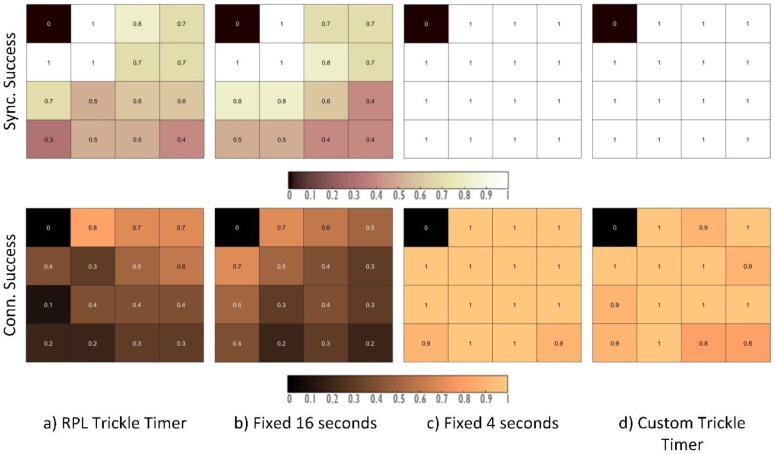
The probability of synchronization and connection success for different EB period configurations.

In the first configuration, Figure 10a, the beacon transmission period is modified following the modifications of the Trickle Timer that is used by RPL and recommended in the Contiki OS implementation, which means that within a few seconds of starting the network, this period has increased considerably. The second configuration, Figure 10b, uses the fixed beacon transmission period that was initially recommended in the standard. This transmission period has been set at 16 s, obtaining results that are similar to those of the Trickle Timer configuration.

The last two configurations, in Figure 10c,d, show how connection probabilities close to 100% are achieved for most nodes in the network. The difference lies in the traffic generated by each configuration that directly affects the energy consumption of the nodes.

**Figure 11:**
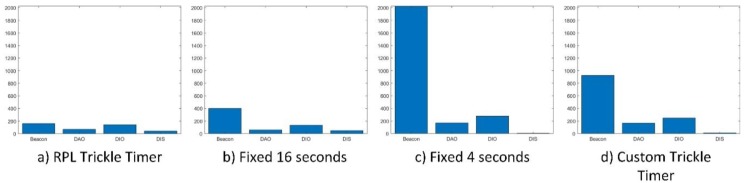
The number of packets for different EB period configurations.

Figure 11 shows the generated traffic for each of the configurations that are depicted in Figure 10. On the one hand, the first two configurations show a rather reduced amount of signalling traffic with respect to the last two configurations. This has been proven to greatly hinder the probability that nodes connect successfully in a short time.

On the other hand, it can be seen how the configuration with a variable beacon transmission period obtains similar connection success results to the fixed transmission period every 4 s, but with a much smaller amount of traffic.

It is important to highlight the influence of the DIS messages that are used by the RPL protocol to request information on the topology. Note that the two configurations that show better results hardly need these control messages to build the topology. This is because these messages were designed to request information about the RPL tree; however, by including the TSCH mechanism, all these messages are discarded as long as there is no synchronization with their time source. Different simulations have been carried out that increase the rate of DIS message transmission (as described in Table 2: Extra Test), and it has been observed that they have a negligible influence on the synchronization mechanism.

**Figure 12:**
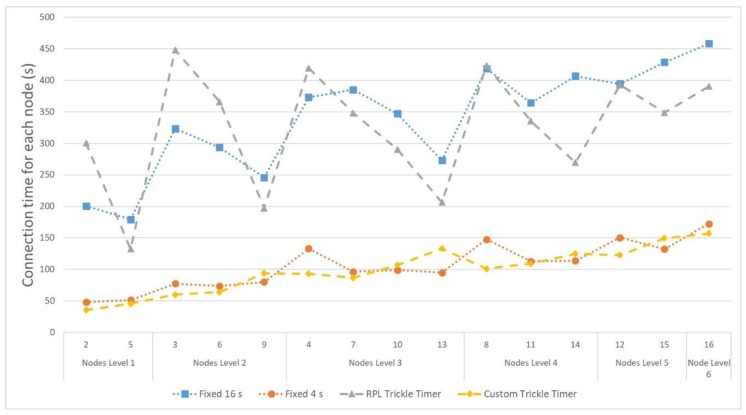
The connection time for different EB period configurations.

Another key result is the maximum connection time that is needed by nodes for the different configurations. Figure 12 shows the connection time for each node and configuration. In the cases where some nodes are not able to connect, a maximum time value of 8 min has been adopted to avoid deviations that are too far from standard values.

Consequently, the first two configurations obtain much higher connection times, mainly because some of the nodes never connect within the first 8 min of simulation. For the two configurations that start sending beacons every 4 s, the time they take to connect the whole network is around 3 min, taking into account that the node at the farthest level (node 16) is at least six jumps from the root node.

**Figure 13:**
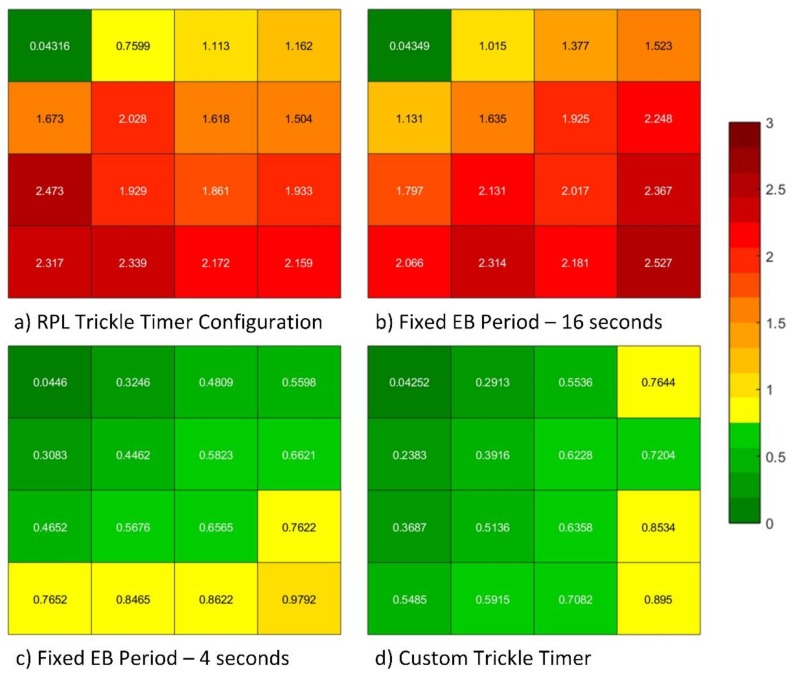
The power consumption for different EB period configurations.

Reducing this connection time will have a great impact on the energy consumption of the nodes. This is because, during the time that they remain scanning, the devices have their radio interface connected persistently, which reduces the autonomy of those nodes that are unable to connect for a long time. Figure 13 shows the energy consumption of the different nodes for each of the configurations.

In configurations that use a lower amount of signalling traffic, Figure 13a,b, energy consumption increases greatly, since, although fewer beacons are sent, the nodes spend more time scanning until they are synchronized.

For the other two configurations, Figure 13c,d, the energy consumption is much lower during the first 15 min, and their results are quite similar. The small variations in consumption levels are mainly due to the reduced statistical mass. If the number of repetitions in the simulations is greatly increased, then these energy consumption levels should tend to the same value in both configurations, since in both configurations the nodes spend 2 min sending beacons every 4 s, and, from that moment, the configuration with the variable period will change to 16 s as proposed by the recommendations.

**Figure 14:**
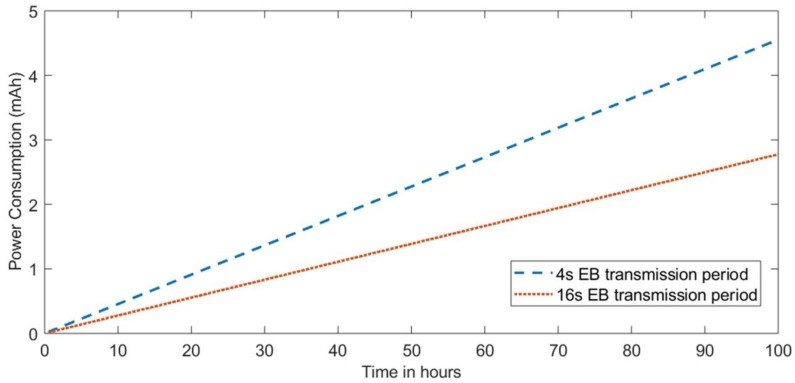
The power consumption for different EB periods over 100 h.

By correlating the consumption results of Figure 13 with the connection times of Figure 12, it can be seen how the energy consumption in this first synchronization phase is mainly affected by the scanning phase, when each node maintains its radio in an active state until it receives a synchronization message. This is why the configuration with the fixed beacons every 4 s and the variable beacon transmission configuration obtain similar results for the first 15 min of operation. Once all of the nodes are connected, the configuration that sends beacons with a fixed period will have a higher energy consumption since it will be transmitting 4 times more messages. Figure 14 shows the evolution of energy consumption for the last two configurations during 100 h, in which one of them maintains its fixed period at 4 s and the other changes the period to 16 s.

### 6.2. Scalability and Topology Considerations

The results and solutions that are presented in this study are influenced by the assumed topology and its implications. Nevertheless, scalability is a common concern when deploying a WSN, as increasing the number of nodes can greatly affect the network’s performance.

Although the proposed mechanism increases the traffic (the number of transmitted packets) with respect to the common solutions, it outperforms these solutions in terms of energy efficiency and connectivity success rate. On the other hand, for all shown mechanisms, (greatly) increasing the number of nodes means increasing the number of EBs in the network, which should enhance the probability of synchronization. Some articles have addressed the issue of scalability by increasing the number of advertiser nodes [21,24,28,29]. However, for a topology in which the nodes are evenly distributed, such as the grid shown in Figure 6, the maximum number of neighbours of a node sending EB messages remains constant and the probability of success is not affected by the quality difference between the links, which allows us to see the improvements in the mechanism clearly. Irregular topologies have not been taken into account because these scenarios could generate nodes that are out of range and other undesirable characteristics that affect the reproducibility and usefulness of the results.

Focusing only on the proposed mechanism, increasing the number of levels in the topology means increasing proportionally the simulation time based on the “2 min per level” limit (for instance, for 10 levels, the simulation would require 20 min). This statement is supported by the fact that the mechanism improves the network joining time of the nodes within the coverage area of a synchronized node. Therefore, nodes belonging to a certain level (i.e., hops to the root node) would require a maximum of 2 min more than the previous level, regardless of the chosen topology.

## 7. Conclusions and Future Works

The presented work analyses the common problems and obstacles for successfully deploying a WSN. This type of network is of interest for their many possible applications and versatility. However, to enable robust operation in industrial scenarios, which are much more demanding, the mechanisms and features that are provided by IEEE802.15.4e and its TSCH mode should be used, such as deterministic, scheduled traffic with channel-hopping possibilities. Nevertheless, different experiments and a deeper analysis have proven that, to achieve a more functional, practical, and optimized network deployment, some more thought has to be directed towards communication configuration parameters and synchronization mechanisms.

The recommendations and implementations that can be found in the standard and popular WSN software distributions fail to optimize critical factors, such as connection success probability, the connection time required, and even energy consumption. In this study, a new configuration, Custom Trickle Timer, has been proposed that introduces significant results and improvements in all three factors with respect to the common configurations.

Still, this work shows interesting room for improvement. A start would be to tweak the variable EB period mechanism so that it changes dynamically in a wider range of values (not limited to 4 and 16 s), and is able to restart when needed by joining new nodes after a long period of operation.

## Figures and Tables

**Table 1:** The configuration’s main parameters.

Physical Parameters	
Min. number of Neighbors	2
Max. number of Neighbors	4
Min. number of hops (node 16)	6
Distance between neighbors	40 m
Max. Simulation duration	15 min

**Table 2:** The configuration parameters that were simulated during the experiments.

	Parameters	Values
**Global Parameters**		
**TSCH**	Scan Duration (TSCAN)	1 s
	Timeslot duration (Tslot)	10 ms
	TSCH Slotframe Schedule Default Orchestra Schedule [10] with multiple slotframes, each one for a particular traffic plane.	EBs: 397 slots in length with only two enabled (TX and RX) RPL: 31 slots in length with only one enabled (Shared)
**RPL**	Trickle Timer Interval Min (Imin)	12
	Trickle Timer Interval Doublings (ID)	8
	Trickle Timer Redundancy Constant (RC)	10
	DIS interval (DISTT)	60 s
**Test 1: Different numbers of channels in steady-state**		
**TSCH**	Number of Channels (NC)	{1,2,4,16} channels
	Number of Scan Channels (NSC)	Equal to NC
**Test 2: Different numbers of scan channels**		
**TSCH**	Number of Channels (NC)	4 channels
	Number of Scan Channels (NSC)	{1,2,4} channels
**Test 3: Different EB period configurations**		
**TSCH**	Fixed EB Transmission Time (EBTT)	{16,4} s
	EB Transmission Time based on Trickle Timer	RPL Trickle Timer
	Custom Trickle Timer	A 4-s period during the first two minutes and then a 16-s period
	Number of Channels and Scan Channels (NC, NSC)	4 channels
**Extra Test: Different DIS interval values**		
**RPL**	DIS interval (DISTT)	{60,45,30,15,5} s

**Table 3:** The power consumption for the different slot types.

*Description*	Time TX Mode (ms)	Current Consumption (TX) (mA)	Time RX Mode (ms)	Current Consumption (RX) (mA)	Power Consumption (mAs)
*Broadcast TX slot power consumption*	4.256	17.4	-	19.7	0.0740544
*Unicast TX slot power consumption*	4.256		2.4 (ACK)		0.1213344
*Broadcast RX slot power consumption*	-		5.452		0.1074044
*Unicast RX slot power consumption*	2.4 (ACK)		5.452		0.1491644
*RX slot without packet reception*	-		2.2		0.04334
*Scan phase*	-		10		0.197

## References

[1] Leitão P., Colombo A.W., Karnoukos S. (2016). Industrial automation based on cyber-pysical systems technologies: Prototype implementations and challenges. Comput. Ind..

[2] Khanafer M., Guennoun M., Mouflah H.T. (2014). A Survey of Beacon-Enabled IEEE 802.15.4 MAC Protocols in Wireless Sensor Networks. IEEE Commun. Surv. Tutor..

[3] (2016). IEC 62591:2016—Industrial Networks—Wireless Communication Network and Communication Profiles—WirelessHART.

[4] (2014). IEC 62734:2014—Industrial Networks—Wireless Communication Network and Communication Profiles—ISA 100.11a.: 2014.

[5] (2015). IEC 62601:2015—Industrial Networks—Wireless Communication Network and Communication Profiles—WIA-PA.

[6] ZigBee Alliance, ZigBee Specification, 2012. https://www.zigbee.org/download/standards-zigbee-specification/.

[7] IEEE 802.15.4-2015 IEEE Standard for Local and Metropolitan Area Networks—Specific Requirements Part 15.4: Wireless Medium Access Control (MAC) and Physical Layer (PHY) Specifications for Low Rate Wireless Personal Area Networks (LR-WPANs). https://ieeexplore.ieee.org/document/7460875.

[8] Borgia E. (2014). The Internet of Things vision: Key features, applications and open issues. Comput. Commun..

[9] De Guglielmo D., Anastasi G., Seghetti A., Gaglio S., Lo Re G. (2014). From IEEE 802.15.4 to IEEE 802.15.4e: A step towards the Internet of Things. Advances onto the Internet of Things. Advances in Intelligent Systems and Computing.

[10] Duquennoy S., al Nahas B., Landsiedel O., Watteyne T. Orchestra: Robust Mesh Networks through Autonomously Scheduled TSCH. Proceedings of the 13th ACM Conference on Embedded Networked Sensor Systems.

[11] Winter T., Brandt A., Hui J.W., Kelsey R., Levis P., Pister K., Struik R., Vasseur J.P., Alexander R.K. (2012). RFC 6550—RPL: IPv6 Routing Protocol for Low-Power and Lossy Networks. Internet Engineering Task Force (IETF) Proposed Standard.

[12] Levis P., Clausen T., Hui J., Gnawali O., Ko J. (2011). RFC 6206—The Trickle Algorithm. Internet Engineering Task Force (IETF) Proposed Standard.

[13] Contiki: The Open Source OS for the Internet of Things: Official Website. www.contiki-os.org.

[14] Stanislowski D., Vilajosana X., Wang Q., Watteyne T., Pister K.S.J. (2014). Adaptive Synchronization in IEEE802.15.4e Networks. IEEE Trans. Ind. Inform..

[15] Chang T., Watteyne T., Pister K., Wang Q. (2015). Adaptive synchronization in multi-hop TSCH networks. Comput. Netw..

[16] Elsts A., Duquennoy S., Fafoutis X., Oikonomou G., Piechocki R., Craddock I. Microsecond-Accuracy Time Synchronization Using the IEEE 802.15.4 TSCH Protocol. Proceedings of the IEEE 41st Conference on Local Computer Networks Workshops (LCN Workshops).

[17] Elsts A., Fafoutis X., Adeyinka A., Piechocki R., Oikonomou G., Duquennoy S., Liñán A., Fàbregas M. Competition: Adaptive Time-Slotted Channel Hopping. Proceedings of the European Conference on Wireless Sensor Networks (EWSN).

[18] Duquennoy S., Elsts A., Nahas A., Oikonomou G. TSCH and 6TiSCH for Contiki: Challenges, Design and Evaluation. Proceedings of the 13th International Conference on Distributed Computing in Sensor Systems (DCOSS).

[19] Watteyne T., Palatella M., Grieco L. (2015). RFC 7554: Using IEEE 802.15.4e Time-Slotted Channel Hopping (TSCH) in the Internet of Things (IoT): Problem Statement. Internet Engineering Task Force (IETF) Informational Document.

[20] Vilajosana X., Pister K., Watteyne T. (2017). RFC 8180: Minimal IPv6 over the TSCH Mode of IEEE 802.15.4e (6TiSCH) Configuration. Internet Engineering Task Force (IETF) Informational Document.

[21] Vogli E., Ribezzo G., Grieco L.A., Boggia G. Fast Join and Synchronization Schema in the IEEE 802.15.4e MAC. Proceedings of the IEEE Wireless Communications and Networking Conference (WCNC).

[22] Vogli E., Ribezzo G., Grieco L.A., Boggia G. (2018). Fast network joining algorithms in industrial IEEE 802.15.4 deployments. Ad Hoc Netw..

[23] Duy T.P., Kim Y. An Efficient Joining Scheme in IEEE 802.15.4e. Proceedings of the IEEE International Conference on Information and Communication Technology Convergence (ICTC).

[24] Duy T.P., Dinh T., Kim Y. (2016). A rapid joining scheme based on fuzzy logic for highly dynamic IEEE 802.15.4e time-slotted channel hopping networks. Int. J. Distrib. Sens. Netw..

[25] Khoufi I., Minet P., Livolant E., Rmili B. Building an IEEE 802.15.4e TSCH network. Proceedings of the IEEE 35th International Performance Computing and Communications Conference (IPCCC).

[26] Khoufi I., Minet P., Rmili B. Beacon Advertising in an IEEE 802.15.4e TSCH Network for Space Launch Vehicles. Proceedings of the 7th European Conference for Aeronautics and Aerospace Sciences (EUCASS).

[27] Kim J.Y., Chung S.E., Ha Y.V. A Fast Joining Scheme Based on Channel Quality for IEEE802.15.4e TSCH in Severe Interference Environment. Proceedings of the IEEE 9th International Conference on Ubiquitous and Future Networks (ICUFN).

[28] De Guglielmo D., Seghetti A., Anastasi G., Conti M. A Performance Analysis of the Network Formation Process in IEEE 802.15.4e TSCH Wireless Sensor/Actuator Networks. Proceedings of the IEEE Symposium on Computers and Communication (ISCC).

[29] Wang L., Reinhardt A. A Simulative Study of Network Association Delays in IEEE 802.15.4e TSCH Networks. Proceedings of the IEEE 18th International Symposium on a World of Wireless, Mobile and Multimedia Networks (WoWMoM).

[30] Vallati C., Brienza S., Anastasi G., Das S.K. (2018). Improving network formation in 6TiSCH networks. IEEE Trans. Mobile Comput..

